# Disparities in Youth Sports and Barriers to Participation

**DOI:** 10.1007/s12178-021-09716-5

**Published:** 2021-10-08

**Authors:** Nirav Kiritkumar Pandya

**Affiliations:** 1 Department of Orthopedic Surgery, University of California San Francisco, San Francisco, CA USA; 2 Department of Pediatric Orthopedic Surgery, UCSF Benioff Children’s Hospital Oakland, 747 52nd Street, Oakland, CA 94609 USA

**Keywords:** Youth sports, Pediatric, Adolescent, Sports medicine, Disparity, Inequity

## Abstract

**Purpose of Review:**

Youth sports participation has shifted from a school-based, seasonal activity to club-based, year-round activity over the past 10–15 years. Single sport specialization has become increasingly common with a concurrent increase in injury and burnout. Paralleling trends seen in other aspects of health care, disparities in regard to participation in youth sports, and subsequent injury treatment exist as well. Recognition of these disparities amongst coaches, parents, and athletes involved in youth sports are essential to promote the short- and long-term health of pediatric and adolescent athletes.

**Recent Findings:**

Multiple barriers exist for youth in regard to sports participation. Youth who come from families without extensive financial means are increasingly finding it difficult to play organized sports, with this trend holding when broken down by insurance status (public versus private). This problem is further exacerbated by the lack of community-based programming in locations where organized (albeit expensive) options do not exist. The lack of athletic trainers increases the divide, as well as the care that injured athletes receive (particularly in public schools within communities of color) is not equivalent to schools with extensive financial resources. Thus, ability to quickly return to play after injury and/or access the health care system is limited. This is further exemplified by inferior outcomes in regard to care for anterior cruciate ligament, meniscus, shoulder instability, and concussions in this population.

**Summary:**

Youth sports participation is laden with multiple disparities. This is unfortunately reflective of historical barriers to opportunities/advancements in multiple other areas of society. These disparities place certain groups of children at an uphill battle not only for participation when healthy, but also returning to participation when injured. Larger structural changes in youth sports are necessary to promote life-long, healthy physical activities for individuals most at risk.

## Introduction

A shift from school-based, sports participation to private, club sports has occurred within the USA. This is the background in which a “pay to play” model has developed excluding families who may not have the economic means to participate. This has been exacerbated by COVID-19 in which adolescent youth have been less physically active [1]. McGuine et al. examined 13,002 adolescent athletes during COVID-19 school closures and found a 74.1% prevalence of depressive symptoms in team sport participants [2•]. This is within a context of a pre-COVID environment in which only 7.9% of middle school and 2.1% of high schools provided daily structured physical education [3].

Preceding this change in activity patterns was a trend toward sports specialization. According to the Aspen Institute, children aged 6–12 years played an average of 2.11 sports in 2011, dropping to 1.87 in 2018 [4]. Single sport specialization is defined as intensive participation in a single sport at the exclusion of the sports [5]. This pattern of sporting participation has been associated with an increased risk of overuse syndromes of the knee, shoulder, and elbow [6••], as well as an increased risk of traumatic knee [7] and elbow injuries [8]. For many parents, the drive for athletic scholarship and/or preferred acceptance to college drives them even in the context of increased injury risk.

Yet, only roughly 7% of high school athletes will play collegiately (of which only 2% will obtain a scholarship), and an even smaller percentage of these athletes will go on to play professionally [9–11]. Only 6 NCAA sports offer full scholarships (football, men’s and women’s basketball, women’s gymnastics, volleyball, and tennis). What has been established is that athletes who play multiple sports are more likely to succeed professionally in sports such as the NBA [12], the NFL [13], and the MLB [14, 15]. In the drive for the rare scholarship, parents, coaches, and athletes not only are increasing their injury risk but also the disparities for sports participation as a byproduct.

## Financial Barriers to Play

In an analysis of NCAA data, fewer than 1 of 7 students playing Division I sports were from families in which neither parent went to college [16]. This may be counterintuitive to those who believe scholarships are reflective of a desire to award those who may not have the economic means to pay for college. Yet, this is reflective of a trend in which significant financial barriers to play exist. Without the ability to participate, only those with robust financial means are able to place themselves in a position to attract the attention of collegiate coaches.

Underlying this is the tremendous financial incentive that the gate-keepers of youth sports, private clubs, have within a 15 billion dollar industry [17]. According to a TD Ameritrade survey, 63% of parents will pay $1200 to $6000 per year for sports participation, with nearly 1 in 5 paying more than $12,000 a year [17]. Furthermore, according to the Aspen Institute, whereas 43% of children in homes earning more than $100,000 were able to play sports, only 22% of children in homes with incomes less than $25,000 participated [4••]. This had led to increased inactivity levels amongst those most at risk of developing long-term health conditions as adults. For families who cannot afford the average $693 per sport per year to participate [4••], limited school-based options (particularly during COVID) has led to a lack of physical activity opportunities due to fees, transportation challenges, and equipment costs [18].

This discrepancy in participation based on income was shown in a recent study by Jayanthi et al. who found that as socio-economic status increased, youth athletes suffered more overuse injuries [19••]. This was postulated to be due to an increased incidence of sports specialization, more hours per week playing organized sports, higher ratio of weekly organized sports compared to free play, and preferential participation in individual sports [19••]. This was further demonstrated by Post et al. who demonstrated that families whose children played club sports spent a median of $1500 dollars (range $500–$3000) per year, had household incomes greater than $100,000, and had bachelor’s degree or higher education [20••]. Barriers to participation for lower socio-economic groups are real within this specific demographic of youth.

With a de-emphasis on school-based sports, the only options available to families are expensive and cost-prohibitive. This decline in participation is particularly striking amongst communities of color [4••]. Hyde et al. found that youth sports participation was highest amongst white males and increased with education/household income [21•]. Even when looking at youth sports participation through the lens of health insurance, Fabricant et al. found that youth with government/Medicaid or lacking health coverage had low levels of physical activity [22]. The barriers to participation are not only financial but also geographic. Communities in which there are higher levels of college attainment are more likely to offer options for club sports such as flag football [23].

Investment in community-based programming, local facilities (green spaces, baseball fields, basketball courts), and funding of school programming are potential strategies that can utilized to improve participation amongst youth in communities of need.

## Disparities in Care Received

Beyond the ability to engage in sports due to financial restraints, many youth experience negative health outcomes in regard to management of their injuries. Even if access to sports exists for many youth, disparities in how their sport-related injuries are managed can prevent return to play. As mentioned above, insurance status plays a large role in determining access to sporting activity. As with other aspects of the health care system, difficulty in accessing care for sports injuries exists with public/no insurance [24]. For many schools, athletic trainers provide both acute/sub-acute medical care for students in need. The ability to treat injuries, guide care, and act as an advocate can provide front line care for sports injuries for many pediatric and adolescent athletes.

Post et al. examined athletic trainer access in Wisconsin and California schools and found that schools located in areas of lower socio-economic status had less access to athletic trainers [25, 26].^.^ This is reflective of a larger trend in which only 67% of schools have access to athletic trainers of which only 35% are full-time [27]. The presence of an athletic trainer has been particularly relevant in the ability to diagnose concussions, an entity in which a disparity exists for young athletes.

Kroshus et al examined concussion diagnoses and found that schools with an athletic trainer had a greater number of athletes diagnosed with a concussion as compared to schools without one [28]. Wallace et al also found similar disparities in regard to concussion knowledge and management based on race and presence of an athletic trainer [29••, 30]. The lack of an athletic trainer also has implications for bassline neurocognitive testing, particularly in public high schools with low-income populations.

Finally, intermittently intertwined with care disparities are the race/gender of the individuals who are providing care in schools. Barriers have been noted for female candidates in obtaining head athletic training positions [31]. Furthermore, the composition of the National Athletic Trainers Association has largely been white with very few trainers of color [32]. This is particularly important as although athletic trainers self-report a high level of cultural competence, they actually operate at a lower level of cultural competence with behaviors that do not mirror cultural awareness or sensitivity based on a study by Marra et al. [33] In at-risk communities (many of which have students of color), an urgent need exists to not only improve the presence of athletic trainers but also the cultural competency of those hired so that treatment for sports injuries can be improved. Barriers to receiving timely medical care can translate into barriers to participation due to time lost to injury.

## Pathology-Specific Disparities

Integral to any discussion in regard to youth sports participation is the recognition of the disparities in care received for the actual injuries sustained due to participation. The lack of timely treatment for sports-related injuries in the pediatric and adolescent population can translate into delayed and/or cessation of participation tied to race and insurance status. This is particularly salient within pediatric sports medicine populations as guardians who have a lower level of education achieved, are non-English speaking, and have public insurance have lower health literary scores [34]. With the appropriate implementation of preventive programs, particularly in lower resourced communities, the physical and financial burden to the health care system through emergency room visits can be lessened for these sports injuries [35].

Bram et al. examined 915 pediatric patients who underwent ACL reconstruction and found that Black/Hispanic patients and those with public insurance had greater delays to surgery, more irreparable meniscus tears, and less physical therapy visits [36••]. This trend was also shown by Patel et al with publically insured patients having a greater incidence of treatment delays, meniscal injuries, and post-op stiffness [37]. Newman et al further showed that ACL reconstruction took twice as long for patients with public versus private insurance [38]. These delays in receiving care increase the risk of meniscal damage (leading to impaired sports performance) as well as prevent safe return to play due to decreased quantity of physical therapy sessions.

The potential long-term outcomes with delay in care have been clearly shown by Williams et al. In a group of 119 patients who underwent ACL reconstruction, the authors found that mean time to presentation was 56 days for the private insurance group and 136 days for the public insurance group [39••]. Patients with public insurance also had more moderate to severe chondral injuries and meniscal tears that required debridement rather than repair. This trend has also been seen in patients with isolated meniscal pathology. Johnson et al. examined pediatric and adolescent patients and found that uninsured/publically insured patients experienced significant delays compared to commercially insured patients [40]. The timing to diagnosis via MRI is also impacted for these knee injuries. Beck et al examined 168 patients who underwent knee MRI and found that the time between injury and MRI was significantly longer for government-insured patients (34 days versus 67 days) as was the time between 1st visit and MRI and MRI order and completion [41].

Shoulder instability also falls within this trend. Hung et al. examined a series of pediatric and adolescent patients with shoulder instability and found that patients with public insurance had to wait 5 times longer for initial evaluation, 4 times longer for MRIs, were twice as likely to have bony pathology, and had post-operative dislocation at higher rates (24.3%) [42].

Finally, concussion care is not spared from disparities in participation, particularly as delayed care can lead to prolonged duration out of participation. Lyons et al. retrospectively examined 11 years of the National Electronic Injury Surveillance System and found that Black children were less likely to be seen in the emergency department for sports-related head injuries as well to be diagnosed with concussions [43]. Furthermore, Wallace et al. examined concussion knowledge and found that white high school athletes had increased concussion knowledge as compared to African-American athletes, although this bridge was shown to lessen when African-American students had access to an athletic trainer [29]. As mentioned above, disparity in relation to treatment of injuries has many layers and can impact return to sport in many ways. Even when diagnosed with a concussion, post-concussion academic support demonstrated disparities with commercial insured, English speaking patient receiving more support, even when follow-up visits were attended [44].

Barriers to participation are complex and multi-factorial. As many youth sports athletes will suffer injuries due to sports specialization, ability to return to play in a timely fashion must be considered. Systemic disparities present within our health care system for certain pediatric and adolescent athletes make it difficult for injured athletes to return to participation and reap the benefits of health sports participation.

## Conclusions

As youth sports participation continues to increase, particularly with skill-based club sports, multiple barriers to participation exist for pediatric and adolescent athletes based on race, socio-economic status, and insurance. This starts at the high financial bar which exists to access club sports and then continues at every level of the health care system. The lack of access to athletic trainers as well as delays in care received for common sports pathologies such as ACL tears and concussions further prevents return to sporting activity after injury, representing a medical barrier to sports participation. A comprehensive program which engages parents, athletes, coaches, community leaders, school administrators, and the health care system will be critical for overcoming these barriers.
